# Supplementary material for: The Importance of Time and Place: Nutrient Composition and Utilization of Seasonal Pollens by European Honey Bees (Apis mellifera L.)
Source: Insects. 2021 Mar 10;12(3):235. doi: 10.3390/insects12030235 (PMC8000538; doi:10.3390/insects12030235)
Supplement: Supplementary file 1 [file insects-12-00235-s001.zip › insects-1067688-supplementary-conversion/Suppl Fig S1-poll consum and digest.docx]

**Spring Fall**

Figure S1. Average (±SE) amount of protein consumed (A) and the proportion of undigested protein measured in the hindgut of 7-day old honey bees that are offspring of queens from California (CAq) or Iowa (IAq) that were fed pollen collected in Arizona (AZp) or Iowa (IAp) in either the spring of the fall. Means with the same letter are not significantly different at the p = 0.05 level as determined by analysis of variance (pollen consumed - spring: F_3,12_ = 24.95, p < 0.0001, fall: F_3,12_ = 15.70, p < 0.0001; undigested pollen - spring: F_3,12_ = 4.99, p = 0.018, fall: F_3,12_ = 1.09, p = 0.39) followed by Fisher pairwise comparisons.

**A**
