# Supplementary material for: The Importance of Time and Place: Nutrient Composition and Utilization of Seasonal Pollens by European Honey Bees (Apis mellifera L.)
Source: Insects. 2021 Mar 10;12(3):235. doi: 10.3390/insects12030235 (PMC8000538; doi:10.3390/insects12030235)
Supplement: Supplementary file 1 [file insects-12-00235-s001.zip › insects-1067688-supplementary-conversion/Table S1 Primer seq .docx]

Table S1. qRT-PCR primer sequences.

| Gene | GenBank accession | Forward primer (5’→3’) | Reverse primer (5’→3’) |
| --- | --- | --- | --- |
| vg | NM_001011578 | CCCACGTTGATCTCCAACTAC | CCGCTTGTCTTGGTCAACTTT |
| Hex70a | NM_001110764.1 | GGGAAATGTCAGCCTTGTTC | ATCAGAGCGTGATTGGCTTT |
| Hex110 | EF625899.1 | GTCCTCAGAATCTTCAACTTC | CAGTTCCTTCAATCAGATCAC |
| actin | AB023025.1 | TGCCAACACTGTCCTTTCTG | AGAATTGACCCACCAATCCA |
