# Supplementary material for: The Importance of Time and Place: Nutrient Composition and Utilization of Seasonal Pollens by European Honey Bees (Apis mellifera L.)
Source: Insects. 2021 Mar 10;12(3):235. doi: 10.3390/insects12030235 (PMC8000538; doi:10.3390/insects12030235)
Supplement: Supplementary file 1 [file insects-12-00235-s001.zip › insects-1067688-supplementary-conversion/Table S2-revised -queen type and pollen type by season.docx]

Table S2. Results from analysis of variance determining the effects of queen and pollen type on pollen consumption, protein digestion, hemolymph protein levels, and hypopharyngeal gland (HPG) size in day-7 workers produced in the spring or fall. Workers were offspring of queens reared and open-mated in Iowa or California (queen type) and fed pollen collected in Iowa or Arizona (pollen type) during spring or fall.

| Response | Factor | Pollen type | F* | p |
| --- | --- | --- | --- | --- |
| total pollen consumed  proportion protein digested  total protein consumed  hemolymph protein  hypopharyngeal gland acini size | queen type  pollen type  queen * pollen type  queen type  pollen type  queen *pollen type  queen type  pollen type  queen * pollen type  queen type  pollen type  queen * pollen type  queen type  pollen type  queen * pollen type  queen type  pollen type  queen * pollen type  queen type  pollen type  queen * pollen type  queen type  pollen type  queen * pollen type  queen type  pollen type  queen * pollen type  queen type  pollen type  queen * pollen type | spring  fall  spring  fall  spring  fall  spring  fall  spring  fall | 33.13  69.26  1.28  96.53  0.95  5.94  1.17  13.12  0.67  3.14  0.01  0.12  3.72  19.45  < 0.001  14.40  0.01  0.24  0.07  23.5  0.63  0.01  7.00  0.17  5.94  5.66  0.19  1.72  1.40  1.07 | <0.0001  <0.0001  0.280  <0.0001  0.349  0.031  0.301  0.004  0.429  0.102  0.919  0.739  0.078  0.001  0.970  0.003  0.946  0.633  0.79  <0.0001  0.45  0.914  0.021  0.685  0.031  0.035  0.670  0.214  0.259  0.321 |

*d.f. = 1,12 for all analyses.
