# Supplementary material for: The Importance of Time and Place: Nutrient Composition and Utilization of Seasonal Pollens by European Honey Bees (Apis mellifera L.)
Source: Insects. 2021 Mar 10;12(3):235. doi: 10.3390/insects12030235 (PMC8000538; doi:10.3390/insects12030235)
Supplement: Supplementary file 1 [file insects-12-00235-s001.zip › insects-1067688-supplementary-conversion/Table S3-revised-same queen diff season.docx]

Table S3. Effects of season and pollen type on pollen consumption, protein digestion, total protein consumed and size of hypopharyngeal gland (HPG) acini in worker honey bees that were offspring of queens emerged and locally mated in either California or Iowa. Bees emerged in spring or fall were fed pollen collected in either Arizona or Iowa during that season.

| Response | Queen type | Factor | F* | p |
| --- | --- | --- | --- | --- |
| total pollen consumed  proportion protein digested  total protein  consumed  hemolymph protein  hypopharyngeal gland acini size | California  Iowa  California  Iowa  California  Iowa  California  Iowa  California  Iowa | Season  Pollen type  Season * pollen type  Season  Pollen type  Season * pollen type  Season  Pollen type  Season * pollen type  Season  Pollen type  Season * pollen type  Season  Pollen type  Season * pollen type  Season  Pollen type  Season * pollen type  Season  Pollen type  Season * pollen type  Season  Pollen type  Season * pollen type  Season  Pollen type  Season * pollen type  Season  Pollen type  Season * pollen type | 41.7  19.6  10.8  32.9  18.5  54.0  9.36  1.16  1.75  50.7  2.67  0.76  33.4  3.38  1.40  8.54  1.98  3.78  29.6  34.8  0.001  6.17  9.00  1.25  7.48  9.89  0.07  1.02  1.02  1.26 | <0.0001  0.001  0.006  <0.0001  0.001  <0.0001  0.01  0.30  0.21  <0.0001  0.128  0.401  <0.0001  0.09  0.26  0.013  0.185  0.076  <0.0001  <0.0001  0.982  0.011  0.011  0.286  0.018  0.008  0.416  0.332  0.332  0.284 |

*d.f. for all analyses are 1,12
