# Supplementary material for: The Importance of Time and Place: Nutrient Composition and Utilization of Seasonal Pollens by European Honey Bees (Apis mellifera L.)
Source: Insects. 2021 Mar 10;12(3):235. doi: 10.3390/insects12030235 (PMC8000538; doi:10.3390/insects12030235)
Supplement: Supplementary file 1 [file insects-12-00235-s001.zip › insects-1067688-supplementary-conversion/Table S4-revised - qn-poll-season.docx]

Table S4. Results from analysis of variance of spring and fall pollen consumption, protein digestion, hemolymph protein levels, and hypopharyngeal gland (HPG) size in day 7 workers produced in the spring or fall that are offspring of either Iowa or California queens. The workers were fed pollen collected in Iowa or Arizona.

| response | factor | F* | p |
| --- | --- | --- | --- |
| total pollen consumed  proportion protein digested  total protein consumed  hemolymph protein  hypopharyngeal gland acini size | Queen type  Pollen type  Season  Queen * pollen  Queen * season  Pollen * season  Queen*pollen*season  Queen type  Pollen type  Season  Queen * pollen  Queen * season  Pollen * season  Queen*pollen*season  Queen type  Pollen type  Season  Queen * pollen  Queen * season  Pollen * season  Queen*pollen*season  Queen type  Pollen type  Season  Queen * pollen  Queen * season  Pollen * season  Queen*pollen*season  Queen type  Pollen type  Season  Queen * pollen  Queen * season  Pollen * season  Queen*pollen*season | 108.65  38.06  74.62  0.26  1.21  53.47  5.50  4.29  3.13  39.76  0.01  1.15  2.51  0.46  18.05  5.31  38.89  0.16  5.21  4.78  0.20  0.07  26.20  19.90  0.14  0.01  1.07  1.03  7.59  6.95  7.95  0.88  1.67  1.73  0.05 | <0.0001  <0.0001  <0.0001  0.630  0.282  <0.0001  0.028  0.049  0.090  <0.0001  0.940  0.294  0.126  0.502  <0.0001  0.030  <0.0001  0.691  0.032  0.039  0.663  0.931  <0.0001  <0.0001  0.710  0.930  0.312  0.321  0.011  0.014  0.009  0.357  0.209  0.201  0.832 |

*d.f. = 1, 24
