# Supplementary material for: The Importance of Time and Place: Nutrient Composition and Utilization of Seasonal Pollens by European Honey Bees (Apis mellifera L.)
Source: Insects. 2021 Mar 10;12(3):235. doi: 10.3390/insects12030235 (PMC8000538; doi:10.3390/insects12030235)
Supplement: Supplementary file 1 [file insects-12-00235-s001.zip › insects-1067688-supplementary-conversion/Table S5- revised-gene expr.docx]

Table S5. Results from Kruskal-Wallis tests comparing median gene expression (2^−ΔΔCt^) in the fat body of 7 day old worker honey bees. The workers were offspring of queens sourced from California or Iowa. and fed pollen collected from Arizona or Iowa in either spring or fall. Separate trials were conducted in spring and fall, and bees were fed pollen collected during the same season as the trial.

| Queen type | Comparison | season | Gene expression | Medians | | H | p |
| --- | --- | --- | --- | --- | --- | --- | --- |
| California  Iowa  California  Iowa  Both queen types | Arizona vs. Iowa pollen  Arizona pollen  Iowa pollen  Arizona pollen  Iowa pollen  California  vs. Iowa | spring  fall  spring  fall  spring vs. fall  spring vs. fall  spring vs. fall  spring vs. fall  spring Arizona pollen  spring Iowa pollen  fall Arizona pollen  fall Iowa pollen | *hex* 70  *hex* 110  *vg*  *hex* 70  *hex* 110  *vg*  *hex* 70  *hex* 110  *vg*  *hex* 70  *hex* 110  *vg*  *hex* 70  *hex* 110  *vg*  *hex* 70  *hex* 110  *vg*  *hex* 70  *hex* 110  *vg*  *hex* 70  *hex* 110  *vg*  *hex* 70  *hex* 110  *vg*  *hex* 70  *hex* 110  *vg*  *hex* 70  *hex* 110  *vg*  *hex* 70  *hex* 110  *vg* | **Arizona**  6.0  3.9  51.2  2.07  0.71  6.2  1.7  0.32  6.3  0.39  0.12  1.92  **spring**  7.02  3.93  51.2  6.0  3.5  21.6  1.38  0.32  6.2  1.70  0.52  3.70  **California**  6.05  3.93  21.65  7.02  3.46  21.65  2.07  0.71  6.25  2.77  1.11  15.2 | **Iowa**  7.00  3.50  21.6  2.77  1.11  13.2  1.40  0.52  3.70  0.67  0.93  5.62  **fall**  2.0  0.7  6.2  2.77  1.1  13.2  0.39  0.12  1.9  0.67  0.93  5.62  **Iowa**  1.70  0.31  13.24  1.38  0.52  3.70  0.39  0.12  1.92  0.67  0.93  5.6 | 0.33  0.33  1.33  0.08  0.08  3.0  <0.0001  0.33  <0.0001  0.75  1.3  1.3  5.33  3.00  5.33  0.08  0.75  5.33  0.75  0.08  0.33  0.33  0.75  1.33  2.08  3.00  5.33  5.33  5.33  5.33  1.33  0.33  0.75  2.08  0.08  1.33 | 0.56  0.56  0.25  0.77  0.77  0.08  1.0  0.56  1.0  0.39  0.25  0.25  0.02*  0.08  0.02*  0.77  0.39  0.02*  0.39  0.77  0.56  0.56  0.39  0.25  0.15  0.08  0.02*  0.02*  0.02*  0.02*  0.25  0.56  0.39  0.15  0.77  0.24 |
